# Glycosylation in the Thyroid Gland: Vital Aspects of Glycoprotein Function in Thyrocyte Physiology and Thyroid Disorders

**DOI:** 10.3390/ijms19092792

**Published:** 2018-09-17

**Authors:** Marta Ząbczyńska, Kamila Kozłowska, Ewa Pocheć

**Affiliations:** Department of Glycoconjugate Biochemistry, Institute of Zoology and Biomedical Research, Jagiellonian University, Gronostajowa 9, 30-387 Kraków, Poland; marta.zabczynska@doctoral.uj.edu.pl (M.Z.); kam.kozlowska@doctoral.uj.edu.pl (K.K.)

**Keywords:** glycosylation, thyroid, thyroid-stimulating hormone, TSHR, thyroglobulin, NIS, pendrin, thyroid autoimmunity, thyroid cancers

## Abstract

The key proteins responsible for hormone synthesis in the thyroid are glycosylated. Oligosaccharides strongly affect the function of glycosylated proteins. Both thyroid-stimulating hormone (TSH) secreted by the pituitary gland and TSH receptors on the surface of thyrocytes contain *N*-glycans, which are crucial to their proper activity. Thyroglobulin (Tg), the protein backbone for synthesis of thyroid hormones, is a heavily *N*-glycosylated protein, containing 20 putative *N*-glycosylated sites. *N*-oligosaccharides play a role in Tg transport into the follicular lumen, where thyroid hormones are produced, and into thyrocytes, where hyposialylated Tg is degraded. *N*-glycans of the cell membrane transporters sodium/iodide symporter and pendrin are necessary for iodide transport. Some changes in glycosylation result in abnormal activity of the thyroid and alteration of the metabolic clearance rate of hormones. Alteration of glycan structures is a pathological process related to the progression of chronic diseases such as thyroid cancers and autoimmunity. Thyroid carcinogenesis is accompanied by changes in sialylation and fucosylation, β1,6-branching of glycans, the content and structure of poly-LacNAc chains, as well as *O*-GlcNAcylation, while in thyroid autoimmunity the main processes affected are sialylation and fucosylation. The glycobiology of the thyroid gland is an intensively studied field of research, providing new data helpful in understanding the role of the sugar component in thyroid protein biology and disorders.

## 1. Introduction

The thyroid gland is crucial to the regulation of metabolism, development and growth, acting via the thyroid hormones triiodothyronine (T3) and thyroxine (T4) [1]. The characteristic molecular system absorbs, concentrates, oxidizes and then incorporates iodine into thyroglobulin (Tg) in thyroid follicles [2]. Tg is the protein backbone for the synthesis of thyroid hormones, which are produced by thyrocytes and secreted into the thyroid colloid [3]. Thyroid peroxidase, also called thyroperoxidase (TPO), contains a heme group in its ectodomain and requires iron for its activity [4]. TPO catalyzes iodine oxidation, iodination of Tg, and extracellular production of monoiodotyrosine (MIT) and diiodotyrosine (DIT) near the apical membrane of thyrocytes. The combination of MIT and DIT forms T3, while T4 consists of two coupled DITs [3,5]. Synthesis of thyroid hormones is regulated by pituitary thyroid-stimulating hormone (TSH), which binds to its thyroid-stimulating hormone receptor (TSHR) on the surface of thyroid follicular cells [6]. All the above-mentioned thyroid proteins (Tg, TPO, TSHR) and thyroid-related proteins (TSH) are *N*-glycosylated; their sugar components are responsible for the proper functioning of glycoproteins [7,8,9,10].

**Figure 1:**
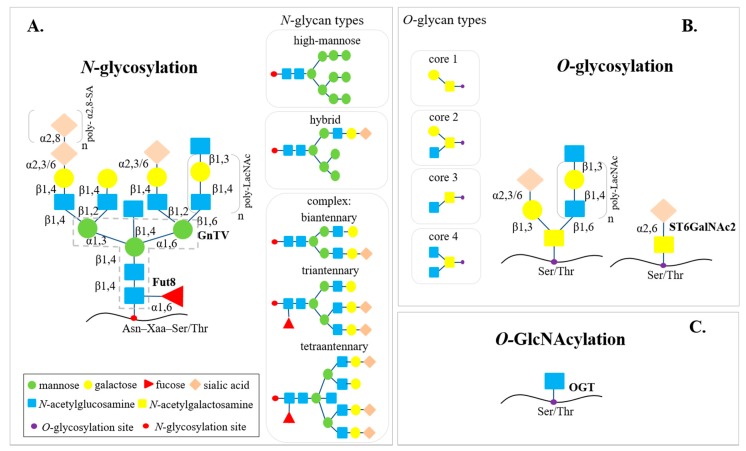
Three post-translational modifications of proteins: *N*-glycosylation, *O*-glycosylation and *O*-GlcNAcylation. (**A**) *N*-oligosaccharides are attached via *N*-glycosidic bonds to asparagine (Asn) in the consensus sequence Asn-Xaa-Ser/Thr (Ser, serine; Thr, threonine; Xaa, any amino acid except proline). In the *N*-glycosylation process, three types of *N*-glycans are created: high-mannose (or oligomannose), hybrid-type, and complex-type bi-, tri- or tetraantennary, which share the same core structure (GlcNAc2Man3, dashed line) and differ in the external part, built of *N*-acetylglucosamine (GlcNAc), galactose (Gal), sialic acid (SA) and fucose (Fuc). Complex-type antennas can be extended with poly-N-acetyllactosamine (poly-LacNAc) chains. (**B**) *O*-glycan structures with mainly cores 1, 2, 3 and 4 are formed in the *O*-glycosylation pathway. *O*-glycans also contain poly-LacNAc chains or are terminated with SA. *O*-oligosaccharides are linked via *N*-acetylgalactosamine (GalNAc) to Ser or Thr in the protein sequence. (**C**) In the *O*-GlcNAcylation process, a single GlcNac is attached to Ser or Thr. Glycosylation processes are catalyzed by different glycosyltransferases, including fucosyltransferase 8 (Fut8), *N*-acetylglucosaminyltransferase V (GnTV), *N*-acetylgalactosamine-specific α2,6-sialyltransferase 2 (ST6GalNAc2) and *O*-GlcNAc transferase (OGT) [13,14,15,16,17,18,19].

Glycosylation is one of the most common post-translational protein modifications (PTMs). Attachment of glycans to a protein is a multistep enzymatic process occurring in the endoplasmic reticulum (ER) and Golgi apparatus. Synthesis of glycans requires transfer of monosaccharides from activated nucleoside triphosphate donors to a sugar acceptor [11]. This process is catalyzed by glycosyltransferases (GTs), which play a key regulatory role in glycosylation; they are responsible for the attachment of sugar moieties to the nascent oligosaccharide structure. Hydrolysis of the glycosidic linkages and release of monosaccharides are catalyzed by glycosidases [12]. Three main types of protein glycosylation have been distinguished: *O*-glycosylation, *O*-GlcNAcylation and *N*-glycosylation, the most abundant PTMs (Figure 1). *N*-oligosaccharides are attached via an *N*-glycosidic bond to asparagine (Asn) in the consensus sequence Asn-Xaa-Ser/Thr (Ser, serine; Thr, threonine; Xaa, any amino acid except proline) during a multistep *N*-glycosylation process. All *N*-glycan structures share the same core sequence Manα1,3(Manα1,6)Manβ1,4GlcNAcβ1,4GlcNAcβ1-Asn (Man, mannose; GlcNAc; *N*-acetylglucosamine). The addition of monosaccharides to the core structures leads to extension of the antennas in the outer part of *N*-glycans. Based on the complexity and composition of monosaccharide residues in the outer part, *N*-glycans have been classified into three groups: oligomannose (high-mannose), hybrid-type and complex-type. Oligomannose glycans contain between five and nine mannoses attached to core GlcNAc residues. The antenna built by GlcNAcβ1,4Gal (Gal, galactose) linked to the *N*-glycan core is characteristic for complex-type *N*-glycans, which are bi-, tri- or tetraantennary structures. The antenna in the outer part of glycans may be modified by the addition of α2,3- or α2,6-linked sialic acid (SA) as a terminal residue, and fucose (Fuc)-linked α1,2 to terminal Gal, and α1,3 or α1,4 to subterminal GlcNAc. Fuc is also present in the core region of *N*-glycans α1,6-linked to the innermost GlcNAc. Hybrid-type oligosaccharides share the features of high-mannose and complex-type *N*-glycans [13,14,15]. *O*-oligosaccharides are attached via *O*-glycosidic bonds to Ser or Thr of proteins and are extended to one of four common forms. A single *N*-acetylgalactosamine (GalNAc) added to the protein chain, known as Tn antigen, is elongated with Gal, giving core 1 *O*-glycans. Modification of Tn antigen with GlcNAc gives a core 3 structure. Further extension of *O*-glycans leads to the formation of branches characteristic for core 2 and core 4 *O*-glycans [16]. *O*-glycan structure can be separated into three regions: the innermost core region described above, a middle region which forms the backbone chain of *O*-glycan, and an outermost region with the highest structural variability [17]. In the case of *O*-GlcNAcylation, a single GlcNAc is added to the Ser or Thr residues (Figure 1) [18,19]. *O*-GlcNAcylation is characteristic for cellular proteins, while most of the surface and secreted as well as some lysosomal proteins are subject to *N*- and *O*-glycosylation [13,20,21].

The modification of proteins by the attachment of oligosaccharides influences protein folding, function, structure and stability, immunological recognition, cell signaling and adhesion [12,22]. Glycans on thyroid proteins play significant roles in Tg transport and hormone synthesis [8], TSH activity [9] and TSH recognition by its receptor [6], as well as iodide transport via sodium/iodide symporter [23] and pendrin [24]. Proper glycosylation of proteins is crucial to the proper functioning of the thyroid gland; changes in GT expression and glycan structure contribute to thyroid disorders [21,25].

The earliest studies on sugar components of the key thyroid proteins were performed in the 1970s and 1980s [26,27]. In 1975 the carbohydrate composition of human TSH subunits was described [26]. At the beginning of 1980s, the crucial role of Tg glycosylation in thyroid hormone production was suggested based on in vitro studies [28]. Then the impact of Tg oligosaccharides on recognition by specific antibodies was shown in the porcine model [29]. These findings were fundamental to further studies aimed at decoding the role of glycans in thyroid proteins that are important in thyrocyte physiology and pathology. Today, glycosylation in the thyroid gland is still a rich field for exploration. Research in the last decade has been aimed at finding serum glycomarkers specific for thyroid autoimmunity and cancers. Some studies have found changes in the glycosylation profile of thyroid proteins during thyroid dysfunction [21,25]. This review summarizes the results of studies on the glycan structures of thyroid proteins, the role of the sugar component in glycoprotein functioning, and alterations of glycosylation in thyroid diseases.

## 2. Glycosylation of Proteins Involved in Thyroid Functioning

### 2.1. Glycosylation of TSH

#### 2.1.1. TSH Protein and Glycan Structure

**Figure 2:**
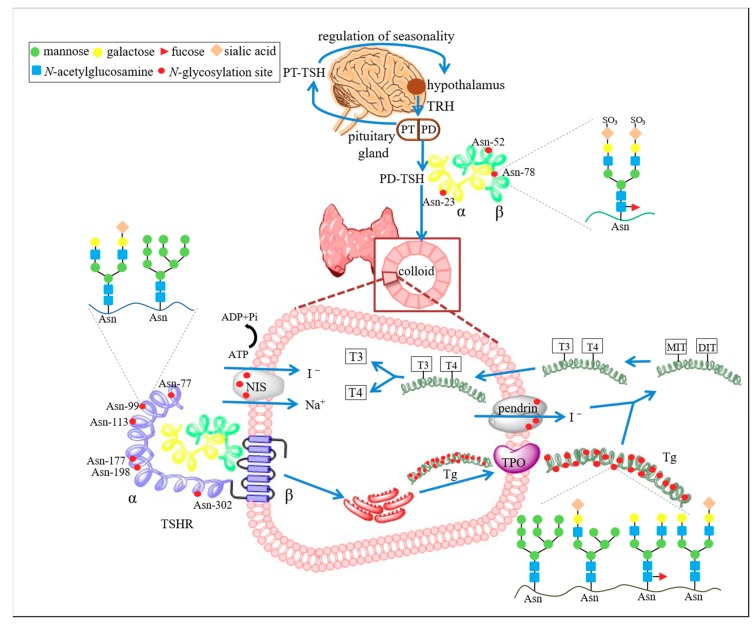
Glycosylation of the key thyroid proteins. Synthesis of thyroid hormones is regulated by the hypothalamus-pituitary-thyroid axis. Thyrotropin-releasing hormone (TRH), produced by the hypothalamus, stimulates the pituitary gland to release thyroid-stimulating hormone (TSH). PD-TSH is secreted by the pars distalis (PD) and PT-TSH by the pars tuberalis of the pituitary gland. PT-TSH binds to the TSH receptor (TSHR) in the hypothalamus and regulates seasonality. PD-TSH binds to TSHR in the cell membrane of thyrocytes and induces signal transduction, resulting in thyroglobulin (Tg) synthesis [31]. Thyroperoxidase (TPO) catalyzes iodine oxidation, iodination of Tg, and production of monoiodotyrosine (MIT) and diiodotyrosine (DIT). The combination of MIT and DIT gives triiodothyronine (T3), while tetraiodothyronine, also called thyroxine (T4), consists of two coupled DITs [3,4,5]. Sodium/iodide symporter (NIS) is responsible for active transport of iodide ions through the thyroid follicular cell membrane into thyrocytes. Pendrin, an anion transporter located in the apical membrane of thyrocytes, is involved in iodide transport from follicular cells into the lumen of follicles [32]. All the above-mentioned human thyroid proteins are *N*-glycosylated and contain different numbers of *N*-glycosylation sites (red dots): TSH–3 (Asn23, Asn52, Asn78) [6], TSHR–6 (Asn77, Asn99, Asn113, Asn177, Asn198, Asn302) [33], Tg–16 (Asn57, Asn179, Asn465, Asn510, Asn729, Asn797, Asn928, Asn1200, Asn1329, Asn1345, Asn1696, Asn1754, Asn1993, Asn2230, Asn2275, Asn2562) [34], NIS–3 (Asn485, Asn497, Asn225) [32,35], pendrin–3 [36]. TSH is abundant in sulfated biantennary *N*-glycans [6]. TSHR contains high-mannose and complex-type structures [33]. High-mannose structures as well as galactosylated, fucosylated, and sialylated hybrid-type and complex-type *N*-glycans have been identified on Tg [34,37,38].

Thyrotropin (TSH) is a glycosylated heterodimer built of noncovalently linked α and β subunits. TSH belongs to the family of glycoprotein hormones, which also includes luteinizing hormone (LH), follicle-stimulating hormone (FSH) and human chorionic gonadotropin (hCG) [30]. The α chain is common to other members of the human glycoprotein hormone family, whereas the β subunit is unique to the TSH molecule. The genes encoding the α and β subunits of human TSH (hTSH) are located on chromosomes 6 and 1, respectively. TSH, produced in the distal part of the pituitary gland, stimulates thyroid cells to synthesize thyroid hormones via TSHR. Human TSH is a 28–30 kDa glycoprotein; the glycan part represents 15–25% of its molecular weight. The amino acid sequence of hTSH contains three potential *N*-glycosylation sites. Two of them are in the α subunit (Asn52, Asn78) and one in the β subunit (Asn23). Each of these oligosaccharide chains are complex-type *N*-glycans (Figure 2).

The heterogeneity of glycan structures results from their different branching, sialylation, core fucosylation and terminal GlcNAc sulfation [6,31,39,40]. The antenna extended on the Manα1,3 arm is terminated with sulfated sugar residues, while the Manα1,6 arm contains additionally α2,3-linked SA as a terminal residue. Differences in glycosylation have also been shown between TSH subunits; α subunit glycans are mainly sialylated and monosulfated, whereas the β subunit contains more disulfated and core-fucosylated structures [40].

#### 2.1.2. Functions of TSH Glycans

The diversity of glycan structures results in many TSH glycoforms, which differ in their bioactivity [6,31]. *N*-glycosylation of the α subunit affects signal transduction after TSHR activation. De-*N*-glycosylation of TSH improves its activity. β subunit glycosylation is important for TSH stability and secretion [41]. Conversion of Asn to glutamine in the β subunit sequence by site-directed mutagenesis significantly reduced TSH production in human embryonic kidney 293 cells cultured in vitro [42]. Binding of de-*N*-glycosylated hTSH to the antibody against the β subunit was mostly failed, whereas a lack of oligosaccharides has little effect on the affinity of the anti-α chain to this subunit. Deglycosylated TSH is fivefold less immunoreactive to the antibody against the β chain than to anti-α. This is due to the presence of the glycosylation-dependent epitopes that determine antigenicity mainly on the β subunit [43].

#### 2.1.3. Glycosylation of Recombinant TSH

The exact protocol for the production of the recombinant human TSH (rhTSH) in Chinese hamster ovary (CHO) cells was described by Cole and colleagues [44]. The molecular mass of the α and β subunits was estimated at 20 and 16 kDa, respectively, based on electrophoretic separation. Analysis of the sugar component showed that rhTSH produced in CHO is more abundant in Man and GlcNAc than pituitary TSH (phTSH). The sialylation level was higher in rhTSH; however, the SA:Gal ratio was comparable between rhTSH and phTSH. Because the plasma clearance rate and toxicology tests performed on monkey and rat showed no treatment-related aberration, rhTSH was suggested as an exogenous source of TSH for treatment of post-thyroidectomy patients [44]. Recombinant hTSH contains sialylated complex-type glycans. Bi- and triantennary structures were present at three *N*-glycosylation sites, while tetraantennary oligosaccharides were attached mainly to Asn23. Fucosylated *N*-glycans were found only at the Asn52 glycosylation site [41]. The glycan composition depends on the primary and secondary structure of proteins as well as on glycosylation processing in the host cells [44,45]. The bioactivity of hormones is determined by their metabolic clearance rate (MCR). The activity and MCR of TSH depend on the *N*-glycan composition of thyrotropin. Desialylation of rhTSH increased its bioactivity in vitro, while in vivo a lack of SA decreased TSH activity in animal model. Resialylation of terminal Gal reversed these effects. Removal of terminal GlcNAc reduced rhTSH activity in vivo, while degalactosylation did not affect the action of thyrotropin [46]. SA-terminated rhTSH glycans differ from pituitary human TSH, which is abundant in sulfated oligosaccharides. The use of several combinations of α and β subunits from rhTSH and phTSH showed that the hybrids containing the desialylated α subunit were more active in vitro. Hybrids with the same α subunit but a different desialylated or fully sialylated β subunit showed similar bioactivity. This means that removing SA from the α subunit but not from the β chain significantly enhances the in vitro activity of highly sialylated rhTSH [10].

It is well described that changes in TSH glycosylation, including sialylation, can alter epitope expression [47,48] and modulate recognition by the specific antibody used in standard TSH immunoassays [47]. Sialylation of rhTSH produced in the CHO cell line differs from phTSH due to a lack of endogenous expression of α2,6-sialotransferase in CHO cells. For this reason, hamster rhTSH was not a reliable assay reference for measurement of the circulating TSH level [48]. A recent study reports that recombinant glycoengineered TSH (rgTSH) is an excellent alternative for rhTSH as a calibrator for TSH measurement [49]. The rgTSH expressed in CHO cells transfected with α2,6-sialotransferase minigene [50] was used as an assay standard to measure TSH level. Comparison of rgTSH, phTSH and rhTSH showed a significant difference in their sialylation profiles. The highest sialylation was found in the rgTSH form. Hypersialylation of rgTSH ensures biological activity and antigenicity similar to circulating TSH. Measurement of TSH in an immunoassay with the use of phTSH, rhTSH and rgTSH as calibrators showed the lowest variation of TSH values in the case of rgTSH. A recombinant glycoengineered variant of TSH is suggested as a new immunoassay calibrator in diagnostic tests [49].

#### 2.1.4. Glycosylation of Naturally Occurring TSH

The mouse pituitary gland produces two types of TSH: PD-TSH in the pars distalis (PD) and PT-TSH in the pars tuberalis (PT). PD-TSH is a classic form of thyrotropin which regulates thyroid hormone synthesis, while PT-TSH binds to TSHR located on mediobasal hypothalamus ependymal cells and activates the expression of the *Dio2* gene, which is responsible for the regulation of seasonality. The PD-TSH and PT-TSH forms also differ in their glycosylation patterns. The molecular mass of PD-TSH (estimated at 37 kDa) is lower than that of PT-TSH (40 kDa). De-*N*-glycosylation reduces the mass of both TSH variants to a 34 kDa protein. Matrix-assisted laser desorption-ionization time-of-flight mass spectrometry (MALDI-TOF-MS) analysis of enzymatically released *N*-glycans has shown that sulfated biantennary glycans are present mainly on PD-TSH, while sialylated tri- and tetraantennary *N*-glycans are characteristic for PT-TSH [31]. Lectin blotting with *Maackia amurensis* agglutinin (MAA), which preferentially recognizes SA α2,3-linked to *N*-acetyllactosamine (LacNAc, disaccharide Galβ1,4GlcNAcβ1,3), and *Phaseolus vulgaris* lectin (PHA-L), which binds β1,6GlcNAc attached to the trimannosyl core [51], confirmed the MS results [31]. The diverse glycan components of PD-TSH and PT-TSH influence the different bioactivities of these pituitary hormones [31].

In human serum, two forms of TSH with different glycosylation profiles have also been identified; the human variants have distinct origins, structures and functions. Free (free-TSH, 44 kDa) and macromolecular (macro-TSH, a complex of TSH and anti-TSH immunoglobulin, 150 kDa) forms were found in sera of patients with hypothyroidism. After peptide *N*-glycosidase F (PNGase F) digestion, the bands of both variants showed the same SDS-PAGE mobility. Lectin affinity demonstrated that nearly half of the free-TSH glycans were recognized by *Concanavalin A* (Con A) specific for α-linked Man, while almost all macro-TSH contained multi-branched *N*-glycans. The distinct glycosylation resulted in altered binding of TSH forms to anti-TSH immunoglobulin [52].

The human thyroid gland is almost completely evolved at 12 weeks of gestation and is capable of thyroid hormone synthesis dependent on maternal TSH. A low level of fetal TSH was first detected at 10 weeks of gestation. The level is higher at 18 weeks of gestation, when the thyroid gland is structurally mature [53]. Glycosylation of TSH in ontogenesis has been studied in rat. The TSH glycosylation pattern changes during ontogenesis in rat. An increase of bi- and multiantennary structures, accompanied by alteration of the oligosaccharide charge resulting from sialylation enhancement, was observed during rat postnatal development [54,55].

Different TSH glycosylation patterns influence in vitro signal transduction through cyclic 3′,5′-adenosine monophosphate (cAMP) and inositol triphosphate (IP3). Human TSHR-transfected CHO and Cos-7 cell lines stimulated by different TSH glycoforms showed significant differences in cAMP and IP3 production. TSH bearing high-mannose structures displayed a higher ability to elevate cAMP and IP3 production than did TSH with biantennary *N*-glycans. Core fucosylation of TSH glycans did not affect cAMP production, but only TSH glycoforms with core Fuc stimulated IP3 synthesis [56].

Removal of the Asn52 *N*-glycosylation site in the β subunit of hTSH resulted in six-fold higher thyrotropin activity expressed in the CHO-K1 cell line as compared with wild-type TSH (wtTSH). Site-directed mutagenesis of the Asn78 and Asn23 residues in the TSH molecule enhanced its activity two- to three-fold, as compared with wtTSH. Also showing increased activity was wtTSH expressed in glycosylation mutants CHO-Lec2 (cells deficient in the CMP-SA transporter, which produce completely desialylated glycoproteins) and CHO-Lec1 (cells without *N*-acetylglucosaminyltransferase I, GnTI, having mainly oligomannose structures in place of complex-type *N*-glycans) [57]. Analysis of glycosylation of human phTSH and circulating TSH using affinity lectin chromatography showed a difference between the glycosylation profiles of phTSH and the circulating form of TSH. Con A specific for Man and ricin, which recognizes Galβ1,4GlcNAc structures, were used in that study. More phTSH was retained on the Con A chromatography column than the circulating hormone, indicating higher content of oligomannose structures on phTSH than on the circulating form. Chromatography with ricin as ligand, performed for fully glycosylated and desialylated TSH, showed lower sialylation of phTSH than circulating TSH. Interestingly, an analysis of fetal sera showed that TSH glycoforms are not sialylated during the fetal period. Desialylated serum TSH from primary hypothyroid patients shows increased binding to ricin, suggesting increased TSH sialylation in this clinical condition as compared with healthy controls [58].

### 2.2. Glycosylation of TSH Receptor

#### 2.2.1. TSHR Protein and Glycan Structure

Thyrotropin receptor (TSHR) is an 84 kDa G protein-coupled 7-transmembrane domain receptor composed of two subunits: an extracellular α chain, which forms the ligand-binding region; and β polypeptide, which encompasses the transmembrane and cytosolic parts of the receptor and is responsible for its signaling. TSHR is encoded by one gene located on chromosome 14 in human and expressed as a single polypeptide cleaved into two subunits and joined by a disulfide bond [59,60]. The ectodomain contains nine leucine-rich repeats (LRRs) and an N-terminal tail, which comprise the binding domain for TSH. Three distinct TSH-binding regions (aa 246–260, 277–296, 381–385) are suggested to form together a complex TSH-binding pocket. Interaction via disulfide bonds between Cys41 and other neighboring Cys in the TSHR α subunit plays a crucial role in high-affinity binding of TSH [53]. The extracellular domain of the human receptor is heavily glycosylated at six *N*-glycosylation sites (Asn77, Asn99, Asn113, Asn177, Asn198, Asn302), and among them Asn113 is unique to hTSHR while Asn177 is specific to mammals (Figure 2).

#### 2.2.2. Functions of TSHR Glycans

Removal of Asn77 or Asn113 results in disruption of TSH binding and inhibition of cAMP synthesis [33,61]. TSHR expressed in the CHO cell line cultured in the presence of tunicamycin, an inhibitor that completely abolishes *N*-glycosylation, showed impaired transport to the cell membrane and loss of its function. Cell surface expression of TSHR produced in CHO-Lec1 (lack of complex-type *N*-glycans) and CHO-Lec2 (SA deficiency) glycosylation mutants was reduced, but the receptor’s ability to bind TSH and synthesize cAMP in response to ligand-binding remained unchanged [62]. Western blot analysis of hTSHR expressed in the CHO-K1 cell line showed two protein bands (120 and 100 kDa). PNGase F digestion revealed that the release of *N*-glycans led to altered migration characteristics of both the upper and lower bands. Treatment with Endo-β-*N*-acetylglucosaminidase H (Endo H, specific for oligomannose *N*-glycans) and neuraminidase (sialidase) indicated that the lower band contains mostly high-mannose *N*-glycans, whereas the glycoform in the upper band is abundant in complex-type structures [63].

TSHR is the main autoantigen in Graves’ disease. The glycans of the TSHR ectodomain play an important role in recognition by autoantibodies. Only the glycosylated variant of the recombinant TSHR ectodomain can bind thyroid stimulatory and blocking antibodies from human serum [64]. Moreover, the α subunit of TSHR, such as thyroglobulin described below, can bind to the mannose receptor (ManR) [65] on the surface of antigen-presenting cells (APC) such as macrophages and dendritic cells. The binding of glycosylated TSHR to ManR mediates phagocytosis and enhances antigen presentation to T cells, which results in initiation and amplification of the immune response [66]. Cleavage of the TSHR polypeptide into α and β subunits also results in shedding of the α subunit from the thyrocyte cell surface. Complex-type *N*-glycans but not immature high-mannose structures were detected on the cleaved α subunit, which must have resulted from cleavage of TSHR on the cell surface. The enzyme responsible for this extracellular shedding has not been identified yet [60]. It is suggested that this truncated highly *N*-glycosylated α subunit contributes to Graves’ disease pathology in genetically susceptible patients via induction and/or maturation of the stimulating anti-TSHR [60,67].

### 2.3. Glycosylation of Thyroglobulin

#### 2.3.1. Tg Protein and Glycan Structure

Thyroglobulin is the most abundant protein in the thyroid gland, and the protein backbone for synthesis of T3 and T4 hormones. The most stable form of Tg is a 660 kDa glycoprotein built of two chains (12S forms), 330 kDa each [68]. In the follicular lumen, besides the soluble 12S form there are also two multimerized variants of Tg (19S dimer, 27S tetramer), present as insoluble globules that serve as a reserve for the production of thyroid hormones [69,70]. Tg contains two regions: the N-terminal domain with the characteristic sequence C-W/Y-C-V-V (ten repeats) and the C-terminal region with high homology to acetylcholinesterase [68].

The Tg molecule is *O*- and *N*-glycosylated [8], and around 10% of its molecular mass is related to oligosaccharides [71,72]. The polypeptide chain of human Tg (hTg) contains 20 putative *N*-glycosylation sites, of which 16 Asn are glycosylated [34]. The main types of hTg oligosaccharides are high-mannose and diantennary complex-type structures (Figure 2) [37]. Eight of the *N*-glycans are fucosylated and galactosylated complex-type, five *N*-glycosylation sites contain high-mannose oligosaccharides, two of them were identified as hybrid- or complex-type without Fuc, and one *N*-glycosylation site was occupied by a variety of *N*-oligosaccharide structures [34]. Tg glycans are highly sialylated [38], with α1,6-linked SA bound preferentially by *Sambucus nigra* agglutinin (SNA) being more abundant than α2,3-SA recognized by MAA [73]. Glycans on Tg in the intrafollicular globules, in contrast to soluble Tg, were not captured by Con A lectin specific for α-linked Man and SNA. This may be due to the covalent cross-links between the Tg molecules stored in a high concentration in the globules, which would reduce the access of lectins to glycan epitopes [69]. Porcine Tg was also reported to contain sulfated *N*-linked carbohydrate chains [74]. The structure of the *O*-glycans identified on Tg is still largely unknown [72].

#### 2.3.2. Functions of Tg Glycans

Glycans are necessary for intracellular and extracellular transport of Tg, protein folding iodination and hormone synthesis, and the proper functioning and immunoreactivity of Tg [72,75]. *N*-glycans located in the N-terminal domain of Tg play a role in iodination of the tyrosine residue and in iodotyrosine coupling. The N-terminal part, which contains the site of hormone synthesis located at Tyr5, also has two potential *N*-glycosylation sites at Asn57 and Asn91. An analysis of two N-terminal peptides—the variant with high-mannose *N*-glycans and the deglycosylated peptide—showed that high-mannose glycosylation resulted in intensive T4 synthesis, while deglycosylation decreased T4 production [8,76]. *N*-glycosylation also influences Tg immunogenicity, as shown by the replacement of high-mannose and biantennary complex-type structures by multiantennary complex-type oligosaccharides that were not found in thyroid Tg [29]. The immunoreactivity of Tg depends significantly on its sialylation; removal of SA increased the immune response against desialylated Tg [77]. Sialylation of Tg *N*-glycans is also important for its transmembrane transporter binding, and influences Tg solubility [72]. Glycans of porcine Tg modulate recognition of anti-thyroid antibodies. Tg secreted by porcine thyroid cells cultured in serum-free medium has a characteristic glycosylation pattern, different from that of thyroid gland thyroglobulin. Tg produced in vitro contained heterogeneous complex-type *N*-glycans and lower content of high-mannose structures, as compared with thyroid-derived Tg [38]. Thyroglobulin produced in vitro showed fourfold lower immunoreactivity with anti-Tg antibody than did thyroid Tg, attributable to differences in glycan composition that influenced antibody binding affinity [29].

The asialoglycoprotein receptor (ASGPR) is a C-type lectin first described on the surface of hepatocytes. ASGPR is responsible for regulation of the serum glycoprotein level; it recognizes and binds asialylated glycoproteins terminated with Gal and GalNAc [78]. Although the ASGPR protein is characteristic for hepatocytes, this receptor is also expressed in thyroid cells. Rat ASGPR is built of two rat hepatic lectin subunits (RHL-1, RHL-2) [79] which contain a carbohydrate recognition domain (CRD) on the extracytoplasmic side. The RHL-1 subunit, located on the apical membrane of thyrocytes, binds poorly sialylated Tg, and this interaction mediates Tg uptake from the colloid, endocytosis, and delivery to lysosomes [80]. Gal and GalNAc used in vitro as RHL-1 inhibitors reduced Tg internalization by 33%. This means that the *N*-glycan-mediated interaction of RHL-1 with Tg is one of the mechanisms initiating Tg internalization but is not necessary for this interaction [80,81]. ASPGR also transfers newly synthesized asialo-Tg from thyrocytes to the follicular lumen. During this transport, Tg is sialylated by membrane-bound sialyltransferase, resulting in detachment of Tg from the asialoglycoprotein receptor and its release to the lumen of the thyroid follicle [70]. The lectin interaction of ASGPR with thyroglobulin, regulated by the level of Tg sialylation, is crucial to Tg transport through the thyrocyte membrane.

### 2.4. Glycosylation of Thyroid Sodium/Iodide Symporter

Sodium/iodide symporter (NIS) is a membrane glycoprotein with molecular mass of approximately 87 kDa, expressed in the salivary glands, gastric mucosa, lactating mammary glands, and most of all in thyroid tissue. NIS is responsible for active transport of iodide ions through the thyroid follicular basolateral membrane into thyrocytes. In lactating mammary gland cells, NIS allows translocation of iodine into milk; this is crucial for nurslings to synthesize their own thyroid hormones [32,35].

NIS contains three potential *N*-glycosylation sites (Asn225, Asn485, Asn497) [32,35], and the *N*-glycans attached to them are important in iodide transport. The use of the single, double and triple mutants of Asn 225, Asn485 and Asn497 generated by site-directed mutagenesis showed that NIS with a low amount or no *N*-glycans remains an active transporter. The triple mutant without *N*-glycans exhibited 50% of wild-type NIS activity [23]. The process of glycosylation is regulated by cAMP. Activation of the cAMP cascade leads to an increase of iodine uptake and translocation to the plasma membrane in thyroid cells. Decreased translocation to the membrane and reduced iodine uptake were found in tunicamycin-treated follicular cells with impaired NIS *N*-glycosylation at the early step of *N*-glycan synthesis [82].

Added to rat thyroid FRTL-5 cells cultured in vitro, KT5823, a staurosporine-related protein kinase inhibitor, was found to increase TSH-induced NIS expression in this cell line [83]. A study by Beyer and co-workers confirmed that KT5823 enhances the NIS protein level in thyroid cells. They observed an increase of two NIS forms (80 kDa fully glycosylated, 60 kDa hypoglycosylated); enhancement of the second glycoform was greater. This increase of NIS level was accompanied by higher radioactive iodide uptake in thyroid cells. In MCF-7 human breast cancer cells, KT5823 up-regulated only the level of the hypoglycosylated 60 kDa form, while the amount of the mature 90 kDa glycoform was reduced by this protein kinase inhibitor. KT5823-treated breast cancer cells also showed lower radioactive iodide uptake. The lower molecular mass of hypoglycosylated NIS suggests that KT5823 has a similar effect on glycosylation to brefeldin A, an inhibitor of protein transport from the ER to the Golgi apparatus. The effect of decreased iodide uptake in experimental KT5823-treated breast cancer cell mutants with single or triple mutations of NIS glycosylation sites (N225Q, N489Q, N502Q, N225Q/N489Q/N502Q) showed that the inhibition of iodide uptake was only partly connected with hypoglycosylation of NIS [84].

### 2.5. Glycosylation of Pendrin

Pendrin is an anion transporter with 11 or 12 transmembrane domains located in the apical membrane of thyrocytes, kidney cells and inner ear cells. Human pendrin is a 780 aa glycoprotein with molecular mass of 110–115 kDa, encoded by the SLC26A4 gene located on chromosome 7. Thyroid pendrin is responsible for iodide transport, while in the kidney pendrin is a chloride and bicarbonate transporter and plays a crucial role in acid-base metabolism [32,85]. Immunohistochemical analysis of thyroid tissue showed that pendrin expression was higher in thyroid tissue specimens from patients with Graves’ disease than in normal thyroid tissue [85].

Pendrin comprises three putative *N*-glycosylation sites in the extracellular domain. De-*N*-glycosylation reduces its molecular mass to 85 kDa [36]. The role of pendrin glycosylation was investigated in vitro in a kidney cell line transfected with cDNA of mouse pendrin containing five *N*-glycosylation sites, two of which (Asn167, Asn172) are glycosylated. Deglycosylation did not influence pendrin expression. The membrane content of a double *N*-glycosylation pendrin mutant (N167A/N172A) was comparable to that of fully glycosylated pendrin. However, removal of the *N*-glycans attached to both Asn167 and Asn172 abolished the intracellular-dependent affinity of pendrin to Cl^−^, HCO_3_^−^ and OH^−^, as was shown in functional assays [24].

## 3. Glycosylation in Thyroid Pathology

The epidemiological statistics show that thyroid diseases, especially autoimmune thyroid diseases (AITDs) and thyroid cancers, are a serious problem [86,87]. According to the American Thyroid Association, around 20 million Americans suffer from some form of thyroid disease [88]. AITDs are the most common causes of thyroid gland dysfunction [89]; their incidence depends on the region, with developed countries showing the highest frequency of cases [90]. The occurrence of autoimmune hypothyroidism is significantly higher in women than in men [91,92]. The most recent American Cancer Society statistics give an estimate of about 53,990 new cases of thyroid cancer in 2018 [93]. The World Health Organization’s International Agency for Research on Cancer reported 52,937 new incidents of thyroid cancer per 100,000 individuals in 2012 [94]. The epidemiological data for 1999 from Silesia Province in Poland shows that 82.04% of the registered thyroid cancer cases were females and only 17.03% were males. The statistics were similar nine years later: 80.97% of the thyroid cancers were diagnosed in women, 19.03% in men. In both surveys most diagnosed cases were papillary thyroid cancer, but there was a significant increase in the incidence of this type of cancer between 1999 and 2008 [95].

### 3.1. Thyroid Cancers

Thyroid cancers (TCs) derived from follicular thyroid cells are classified as well-differentiated papillary and follicular thyroid carcinomas, as well as poorly differentiated and anaplastic thyroid carcinomas. Papillary thyroid carcinoma (PTC) is the most common type of thyroid cancer, with 80–85% frequency; follicular thyroid cancer (FTC) occurs less frequently (10–15% of TC), and the rarest is anaplastic thyroid cancer (ATC) with occurrence lower than 5% of TC [96,97]. Most TCs are characterized by genetic abnormalities; among them, mutations of genes encoding proteins of the mitogen-activated protein kinase (MAPK) signaling pathway are well described [98].

#### 3.1.1. Alterations of Glycan Profiles in TC

Changes in the expression of key GTs, and the glycosylation profile, including sialylation and fucosylation, complex-type *N*-glycan branching, the presence of bisecting GlcNAc, as well as the content and structure of poly-LacNAc chains, have been reported in many types of cancers [99], including TCs [25]. Alterations of the glycan profile in TCs have been observed in thyroid tissue sections and in thyroid cells cultured in vitro. Moreover, the expression of galectins specific for β-galactose or LacNAc disaccharide was altered in TCs [100,101].

##### Sialylation

Sialylation of thyroid proteins is important to thyroid cancer progression. Changes in sialylation were analyzed at gene level for sialyltransferase (ST) expressed in TC cells. Sialyltransferases α2,8 (ST8Sia) belong to a group of enzymes that catalyze SA linking to another sialic acid through an α2,8-glycosidic bond and the formation of polysialylated glycan chains [102]. The expression of different variants of *ST8Sia* was evaluated in FTC human tissue, the highly invasive FTC-238 cell line, the FTC-133 non-invasive thyroid cancer cell line, normal thyroid tissue and the normal thyroid cell line Nthy-ori 3-1. *ST8Sia4* gene expression decreased in FTC and FTC-238, as compared with FTC-133 and normal thyroid cells. In contrast, the *ST8Sia6* variant was up-regulated in invasive FTC-238 cells. Silencing of *ST8Sia4* in FTC-133 increased cell proliferation, mobility and colony formation ability, while overexpression of *ST8Sia4* in FTC-238 inhibited cell proliferation and decreased colony formation ability and migration. The expression of miRNA146a and miRNA146b, negative regulators of *ST8Sia4*, was found to be increased in FTC-238, as compared to FTC-133 and Nthy-ori 3-1 cells. PI3K/Akt/mTOR signaling is partially involved in *ST8Sia4* suppression induced by miRNA146a/b [103]. The expression of another sialyltransferase, *ST6GalNAc2*, which mediates the transfer of SA to terminal GalNAc and SA binding via α2,6-linkage, was higher in FTC-238 invasive cells than in FTC-133 non-invasive cells and silencing of *ST6GalNAc2* in the FTC-238 cell line reduced its invasive ability. A xenograft of FTC-238 cells with silenced *ST6GalNAc2* showed lower tumor volume in mice, as compared with the control FTC-238 xenograft. Overexpression of *ST6GalNAc2* in the FTC-133 non-invasive cell line enhanced its invasive ability and increased the tumor volume in xenograft mouse [104].

Lectin histochemical staining of SA in tissue specimens of four human thyroid carcinomas showed that cancer transformation of thyroid follicular epithelial cells to PTC and FTC is associated with an increase of sialylation [105,106]. Changes in sialylation in thyroid diseases involve the Tg molecule. Decreased content of SA in Tg glycans was observed in patients with TCs and Graves’ disease [107,108]. Impaired sialylation shortened the half-life of Tg [107].

##### Fucosylation

Different types of human TC analyzed in clinical biopsies have characteristic modes of expression of the *FUT8* gene, which encodes α1,6-fucosyltransferase (Fut8), responsible for attachment of Fuc by α1,6-glycosidic bonds to the innermost GlcNAc in core glycan structures [25,109]. An immunohistochemical method used to detect Fut8 in human TCs showed the strongest staining in PTC, as opposed to normal follicles, and no differences between FTC and normal thyroid tissue. Elevated Fut8 expression was associated with increased PTC tumor size and metastasis to lymph nodes [25,110]. The latest study demonstrated that TC progression is also accompanied by diverse expression of α-l-fucosidase *FUCA1*, a lysosomal enzyme that removes Fuc from oligosaccharides. The mRNA expression of *FUCA1* (assessed by real-time PCR), as well as the presence (immunohistochemical staining and Western blotting) and activity of this enzyme in different TCs, showed reduced amounts of FUCA1 on gene and protein levels in ATC, as compared with PTC, normal human thyroid tissues and cell lines [109]. The lower *FUCA1* expression was accompanied by higher *FUT8* levels in ATC than in PTC, resulting in stronger fucosylation in anaplastic thyroid cancer. The low level of *FUCA1* was suggested to be related to the aggressiveness of ATC more than to tumor growth. In view of the latest results demonstrating that *FUCA1* is a downstream target of the p53 gene, and reports indicating that ATC is usually characterized by a mutated form of p53 while PTC carries wild-type p53, it has been suggested that p53 regulates *FUCA1* gene expression in TCs [111].

##### *O*-GlcNAcylation

Altered glycosylation in TCs has also been observed for *O*-GlcNAcylation, the post-translational modification of nuclear or cytoplasmic proteins by binding of a single GlcNAc to Ser or Thr. Two enzymes are involved in this process: *O*-GlcNAc transferase (OGT), responsible for addition of GlcNAc; and *O*-GlcNAc hydrolase (OGA), which removes GlcNAc [18]. OGA activity is higher in human surgical specimen of TCs than in non-neoplastic tissue samples. *O*-GlcNAc-modified proteins in thyroid cells were found mainly in the nuclear fraction. Nuclear proteins are less *O*-GlcNAcylated in thyroid tumor cells than in non-neoplastic tissues [112]. Inhibition of OGA enzyme by PUGNAc, a GlcNAc analog, or silencing of OGA mRNA, increased the *O*-GlcNAc level in the ATC 8305C cell line. Down-regulation of OGA activity enhanced the phosphorylation of Akt kinase induced by insulin-like growth factor 1 (IGF-1); it resulted in increased cell viability and proliferation [113]. The importance of OGT in TC progression has been investigated in ATC cell line variants with overexpression of OGT, inhibition of OGA, and OGT silencing. Both OGT overexpression and OGA inhibition increased thyroid cell proliferation, while the opposite effect, attenuated cell proliferation, was observed in OGT-silenced cells. Higher *O*-GlcNAc level was associated with more intensive colony formation and thyroid cell mobility [114].

##### Other Types of Glycan Modification in Thyroid Cancer

Alterations of Tg glycosylation in TC progression also involve other types of monosaccharides. Lectin affinity testing with *Lens culinaris* agglutinin (LCA), specific for Man and glucose residues, was useful in differentiating serum Tg from patients with benign and metastatic TCs. The LCA-positive Tg fraction was significantly lower in TC patients with lymph node metastases than in those with benign thyroid tumors [115].

Lectin staining of human histological sections of different TC tissues demonstrated that poly-LacNAc chains are preferentially recognized by these lectins in PTC samples, and to a lesser extent in FTC and other types of TC. Heterogeneous poly-LacNAc chains were identified in PTC: long and short unbranched linear-type and highly branched chains [116].

*N*-acetylglucosaminyltransferase V (GnTV) catalyzes β1,6 branching of complex-type *N*-glycans via transfer of GlcNAc from uridine 5′-diphosphate-GlcNAc (UDP-GlcNAc, nucleotide sugar donor) to position 6 of α1,6 Man in the core structure of *N*-glycans. Up-regulation of GnTV expression was observed in human FTC and was positively correlated with the expression of matriptase, a tumor-associated transmembrane protease. β1,6-branching of matriptase *N*-glycans delayed the degradation of this protein and increased its prometastatic activity [117].

The diffuse sclerosing variant of papillary thyroid carcinoma (DSPTC) is characterized by abundant lymphocytic infiltrates. The mechanism of lymphocytic infiltration within malignant thyroid tissue remains unknown. High endothelial venule (HEV)-like vessels are a potential channel of lymphocyte recruitment. They enable efficient lymphocyte trafficking and have been found in DSPTC tissue. Lymphocyte trafficking via HEV-like vessels in PTC occurs in the same way as in secondary lymphoid organs via HEV. Lymphocyte-endothelial cell interactions mediated by adhesion molecules are required in both physiological and cancer tissues. Glycosylated receptors play a crucial role among the surface proteins responsible for lymphocyte-endothelial interactions. Immunohistochemical analysis of glycans expressed on HEV-like vessels in surgical specimens of human DSPTC showed the presence of 6-sulfo-LacNAc, sialyl-Lewis^X^ (sLe^X^, SA2,3Gal1,4(Fuc1,3)GlcNAc) and sialylated 6-sulfo-LacNAc, which serve as glycoepitopes for l-selectins on lymphocytes. This means that glycan-covered HEV-like vessels can take part in the initial step of lymphocyte accumulation in DSPTC [118].

#### 3.1.2. Glycosylation of Specific Proteins in TC

##### Glucose Transporter 1

There are interesting results on the glycosylation of glucose transporter 1 (Glut1) in TCs. The Glut1 molecule contains 12 membrane-spanning domains and a single potential *N*-glycosylation site (Asn45) located on the first extracellular loop. *O*-glycan structures were also detected on this membrane protein. Glut1 expression is higher in ATC than in normal tissue, and the glycosylation of Glut1 differs between cancer and normal thyroid cells. Glycosylation of Glut1 in thyroid cells isolated from patient with ATC significantly influences active glucose input to cells [119]. An increase of sugar uptake stimulates the metabolism and expansive activity of cancer cells [120]. De-*N*-glycosylated Glut1 showed reduced glucose transport across the plasma membrane to 50% of that of the fully *N*-glycosylated form. On the other hand, inhibition of the early stage of *N*-glycosylation process and accumulation of high-mannose and hybrid-type *N*-glycans did not have a negative impact on glucose transport. *O*-linked glycans are also important to Glut1 function; blocking *O*-glycosylation decreased glucose transport, as in the case of de-*N*-glycosylation [119].

##### Serum Proteins

Glycosylation of serum proteins has been found to undergo changes during the progression of different cancers [121,122], among them thyroid carcinomas [21,123]. Modified glycosylation of serum IgG seems to be a good glycomarker of many diseases, including TC. In thyroid patients there were significantly fewer agalactosylated structures with core Fuc (G0F) as well as agalactosylated core-fucosylated structures with bisecting GlcNAc (G0FN) attached to the Fc fragment of serum IgG1 than in healthy individuals. In thyroid cancer patients the reduction of agalactosylated and core-fucosylated glycans on IgG1 was accompanied by an increase of sialylated structures, mainly G2S [123]. Many types of membrane or secreted glycoproteins have been shown to be up-regulated in thyroid carcinomas, among them mucins and adhesion proteins [21].

### 3.2. Glycosylation in Hypothyroidism and Hyperthyroidism

Hypothyroidism and hyperthyroidism are mainly the results of pathological processes within the thyroid gland and are among the primary thyroid diseases. Rare cases can also arise from disorders of the hypothalamus or pituitary gland or from peripheral causes. The most common thyroid dysfunctions are caused by thyroid autoimmunity, including Hashimoto’s thyroiditis (HT) and Graves’ disease (GD) [124]. AITD is characterized by immunogenicity of the major thyroid antigens (Tg, TSHR and TPO), and a high degree of glycosylation is one of the causes of immunogenicity [125].

Results on *N*-glycosylation of anti-Tg immunoglobulin (IgG) are among the few obtained for glycosylation in AITD. A higher level of serum anti-Tg is the main marker of AITD, especially in HT patients. *N*-glycosylation of anti-Tg from patients with HT, with GD and with PTC was investigated using ELISA lectin assays. Among the three groups, Hashimoto anti-Tg samples had the lowest core fucosylation. There were no observed differences in the galactosylation and SA content of anti-Tg IgG between AITD and PTC patients [126]. Further study showed higher content of Man, terminal SA, core Fuc and Galβ1,4GlcNAcβ1,2Man glycans in anti-Tg IgG isolated from HT patients than in healthy individuals [127]. Our recent study of three different European cohorts also showed decreased IgG core fucosylation in AITD patients. The reduced core fucosylation of IgG was inversely related to the level of anti-TPO. Using *Ulex europaeus* agglutinin (UEA I) specific for α1,2-linked Fuc, we also detected lower content of Fuc in the glycan antennas of peripheral blood mononuclear cells (PBMCs) from HT patients. We did not find shared genetic variance between AITD and glycosylation [128].

Grave’s disease, the most common cause of hyperthyroidism, is characterized by goiter and ophthalmopathy, and is also associated with thyrotoxicosis [129]. Autoimmune processes are triggered by the stimulating autoantibody against TSHR, which mimics the action of TSH. Activation of TSHR results in increased secretion of T3 and T4, and hypertrophy of thyroid follicular cells. Apart from stimulating anti-TSHR, blocking antibodies are also detected in 25–75% of GD patients. The different biological effects of stimulating and blocking anti-TSHR depend on the epitope of the TSHR molecule to which the antibody binds [90,130,131]. GD affects humans but not animals; this may be due to the difference in *N*-glycosylation of the TSHR α subunit, which contains six potential *N*-glycosylation sites in humans while in animals the α subunit contains fewer *N*-glycans. Even great apes, closely related to humans, have five *N*-glycan motifs in TSHR sequence. It has been suggested that more intensive *N*-glycosylation could play an important role in breaking self-tolerance in humans, leading to development of GD [129]. This hypothesis was verified in the BALB/c murine model of hyperthyroidism. Mouse TSHR also has one *N*-linked glycan less than the human receptor. Mice were immunized with an adenovirus expressing the mouse or human TSHR α subunit, which share approximately 87% amino acid homology. The immune response was developed in 47% more transgenic BALB/c mice immunized with a low level of the hTSHR α subunit than in mice injected intramuscularly with a high dose of the adenovirus expressing the mouse TSHR α subunit (none of the mice produced anti-TSHR). BALB/c mice with knock-out of the *TSHR* gene showed a much stronger immune response when they were immunized with the adenovirus expressing the more highly *N*-glycosylated human TSHR α subunit than those immunized with the adenovirus expressing the less *N*-glycosylated mouse TSHR α subunit [129,132,133,134].

The thyroid tissue of patients with GD showed significantly higher total activity of sialyltransferases and higher mRNA level of sialyltransferases 1 (*ST6Gal1*) and 4 (*ST3Gal4*) than did control nontumorous tissue. A positive correlation between *ST6Gal1* expression and TSH receptor antibody level was also observed. Increased *ST6Gal1* and *ST3Gal1* mRNA was associated with elevated SA content in thyroid gangliosides. Sialylation of thyroid gangliosides was highest in GD samples; the profile of these lipids did not differ between GD, toxic and nontoxic thyroid nodules, and nontumorous tissue groups [135].

Glycosylation changes have also been observed in congenital hypothyroidism (CH) not connected with HT [53]. Sialylation of glycans attached to both TSH subunits was up-regulated in rats with primary CH [39]. Further research on the CH rat model confirmed the enhanced sialylation by showing that the level of TSH with sialylated multiantennary *N*-glycans secreted in vitro by pituitary explants from CH rodents was higher than in control animals [136,137]. Changes in TSH glycan sialylation affect the MCR (described above).

Autonomously functioning thyroid nodules (AFTN), connected mostly with constitutively activated mutated TSHR, are a common cause of hypothyroidism. A comparative analysis of gene expression in AFTN and normal surrounding tissue indicated 20–40 genes with changed expression in AFTN, including up-regulated sialyltransferase 1 (ST6Gal1) [138].

Studies have demonstrated that TSH regulates the level of GTs in the thyroid. TSH stimulated the expression of various GTs, including sialyltransferase, in porcine cells cultured in vitro [135,139], and up-regulated the sialylation of Tg by shifting the terminal monosaccharide from Gal to SA. In in vitro studies, tri- and tetrasialylated glycans were detected on Tg. TSH stimulation mainly increased mono- and disialylated oligosaccharides of Tg [38]. Another study showed reduced α2,6-sialylation and unchanged α2,3-sialylation of membrane and secreted Tg in response to TSH stimulation in the RTL-5 hormone-responsive rat thyroid cell line [140]. Treatment of rats with propylthiouracil, used in therapy of hyperthyroidism to enhance endogenous TSH, increased the levels of mannosyltransferases and galactosyltransferases in the thyroid, while rats treated with thyroxine to suppress TSH production showed decreased levels of these glycosyltransferases and *N*-acetylglucosaminidase 141]. Hyposialylated Tg resulting from reduced sialyltransferase activity affected iodotyrosine coupling and transport of Tg into the follicular lumen in a patient with congenital goiter and hypothyroidism [141]. Due to the involvement of all the above enzymes in the synthesis of glycans attached to Tg, this TSH-regulated glycosylation may play a role in T3 and T4 production in rat [142]. Interestingly, increased sialylation in hypothyroid animals resulted from a higher α2,3-sialyltransferase mRNA level in thyrotrophs, related to SA content in the TSH molecule [143].

The glycosylation pattern of hTSH from sera of hypothyroid patients differs from that of euthyroid donors. An increase of TSH glycoforms with terminal Gal and SA was observed in individuals with subclinical hypothyroidism and overt primary hypothyroidism. Serum TSH was elevated after administration of pharmacological doses of thyrotropin-releasing hormone (TRH) to patients with subclinical and overt primary hypothyroidism, but TRH treatment did not influence the amount of sialylated or terminally galactosylated TSH isoforms [144]. Analysis of TSH glycans in sera of hypothyroid patients also showed decreased core fucosylation, as compared with healthy donors [41,145]. Higher levels of terminal SA and Gal were observed in patients with resistance to thyroid hormone T3; this is a rare genetic disease resulting from a mutation of the thyroid hormone receptor β (TRβ1). A defect in the T3-binding domain of TRβ1 reduces the interaction of thyroid hormone with the receptor and leads to hypothyroidism [9].

Thyroid hormones T3 and T4 regulate metabolism, growth and development by controlling gene expression. Among more than two thousand liver genes, 55 genes have been found to be regulated positively and negatively by T3. In the hypothyroid mouse model, 41 liver genes, including genes encoding α2,6- and α2,3-sialyltransferases, were down-regulated in response to thyroid hormone treatment [146].

## 4. Conclusions

Since the early research of the 1970s it has been known that most of the thyroid gland and thyroid-related proteins are glycosylated and that sugar chains are an important part of thyroid glycoproteins, significantly regulating protein function. The more comprehensive approaches and high-throughput technology used nowadays enable researchers to make detailed analyses of monosaccharide composition, to determine the glycosidic bond types in glycan structures, their conformation and occupation of potential glycosylation sites, and to make functional assessments of the roles of glycans. The results of interdisciplinary research in glycobiology and endocrinology confirm that oligosaccharides are critically involved in the control of thyroid functioning in the physiological state, and that changes in protein glycosylation profiles lead to thyroid pathologies, including thyroid carcinogenesis and autoimmunity.

## Abbreviations

aa	amino acids
AFTN	autonomously functioning thyroid nodules
AITD	autoimmune thyroid disease
Akt	protein kinase B (PKB)
ASGPR	asialoglycoprotein receptor
APC	antigen-presenting cell
ATC	anaplastic thyroid cancer
cAMP	cyclic 3′,5′-adenosine monophosphate
CMP	cytidine monophosphate
CH	congenital hypothyroidism
CHO	Chinese hamster ovary cell line
CHO-Lec	glycosylation mutant of CHO
Con A	*Concanavalin A* lectin
CRD	carbohydrate recognition domain
DIT	diiodotyrosine
DSPTC	diffuse sclerosing variant of papillary thyroid carcinoma
Endo H	endo-β-*N*-acetylglucosaminidase H
ER	endoplasmic reticulum
FSH	follicle-stimulating hormone
FTC	follicular thyroid cancer
Fuc	fucose
FUCA	α-l-fucosidase
Fut8	α1,6-fucosyltransferase
G0F	agalactosylated IgG *N*-glycans with core fucose
G0FN	agalactosylated IgG *N*-glycans with bisecting GlcNAc and core fucose
G2S	bigalactosylated IgG *N*-glycans with sialic acid
Gal	galactose
GalNAc	*N*-acetylgalactosamine
GD	Graves’ disease
GlcNAc	*N*-acetylglucosamine
Glut1	glucose transporter
GnT	*N*-acetylglucosaminyltransferase
GT	glycosyltransferase
hCG	human chorionic gonadotropin
HEV	high endothelial venule
HT	Hashimoto’s thyroiditis
IGF-1	insulin-like growth factor 1
IgG	immunoglobulin
IP3	inositol triphosphate
LacNAc	*N*-acetyllactosamine
LCA	*Lens culinaris* agglutinin
LH	luteinizing hormone
LRR	leucine-rich repeat
MAA	*Maackia amurensis* agglutinin
MALDI-TOF-MS	matrix-assisted laser desorption-ionization time-of-flight mass spectrometry
Man	mannose
ManR	mannose receptor
MAPK	mitogen-activated protein kinase
MCR	metabolic clearance rate
MIT	monoiodotyrosine
mTOR	mammalian target of rapamycin
NIS	sodium/iodide symporter
OGA	*O*-GlcNAc hydrolase
OGT	*O*-GlcNAc transferase
PBMC	peripheral blood mononuclear cell
PD-TSH	pars distalis thyrotropin
PHA-L	*Phaseolus vulgaris* lectin
phTSH	pituitary human thyrotropin
PI3K	phosphatidylinositol-3-kinase
PNGase F	peptide *N*-glycosidase F
PTC	papillary thyroid carcinoma
PTM	post-translational protein modification
PT-TSH	pars tuberalis thyrotropin
RHL	rat hepatic lectin
rhTSH	recombinant human thyrotropin
SA	sialic acid
Ser	serine
sLe^X^	sialyl-Lewis^X^
SNA	*Sambucus nigra* agglutinin
ST	sialyltransferase
ST3Gal4	sialyltransferase 4
ST6Gal1	sialyltransferase 1
ST6GalNAc2	*N*-acetylgalactosamine-specific α2,6-sialyltransferase 2
ST8Sia	α2,8-sialyltransferase
T3	triiodothyronine
T4	thyroxine
TC	thyroid cancer
Tg	thyroglobulin
Thr	threonine
TPO	thyroperoxidase
TRβ1	thyroid hormone receptor β
TRH	thyrotropin-releasing hormone
TSH	thyroid-stimulating hormone
TSHR	thyrotropin receptor
UDP	uridine 5′-diphosphate
UEA I	*Ulex europaeus* agglutinin I
